# The relationship between square dance exercise and quality of life in middle-aged and older adults: chain mediated effects of negative emotions and attitudes toward aging

**DOI:** 10.3389/fpubh.2024.1470624

**Published:** 2024-10-22

**Authors:** Ting Ouyang, Yujia Qu, Xinyu Du, Ming Fan, Yan Wang

**Affiliations:** ^1^School of Sports Science, Beijing Sport University, Beijing, China; ^2^Department of Sports, University of Science and Technology Beijing, Beijing, China; ^3^School of Education, Beijing Sport University, Beijing, China; ^4^Department of Sports, North China Electric Power University, Beijing, China; ^5^School of Art, Beijing Sport University, Beijing, China

**Keywords:** square dance exercise, quality of life, negative emotions, attitudes toward aging, middle-aged and older adults

## Abstract

**Background:**

With the continuous development of society and the economy, population aging has become an inevitable global development trend, triggering a series of social problems and having a more serious impact on the physical and mental health of middle-aged and older adults. Physical exercise is one of the key factors for improving the overall health and quality of life of individuals, but the role of square dance exercise and the quality of life of middle-aged and older adults have not yet been clarified.

**Purpose:**

This study aimed to investigate the associations between square dance exercise and the quality of life of middle-aged and older adults and its mechanism of action, including its effects on physical health and mental health, with negative emotions and attitudes towards aging as mediating variables.

**Method:**

This study adopted a cross-sectional research method, using the Physical Activity Rating Scale (PARS-3), Depression-Anxiety-Stress Scale in Simplified Chinese (DASS-21), Attitudes toward Aging Questionnaire (AAQ), and 12-item Short Form of the Health Survey (SF-12), and launched an online survey on the QuestionStar platform from August–September 2023 for 4,636 middle and older adults aged 45–74 years. The survey results were analyzed via Pearson correlation analysis via SPSS 27.0 and structural equation modeling analysis via Mplus 8.3 to test the model fit and mediation effects.

**Results:**

Square dance exercise was significantly correlated with negative emotions, attitudes towards aging, and quality of life (*r* = −0.042–0.878, *p* < 0.01). Square dance exercise significantly and positively predicted quality of life (*β* = 0. 202, *p* < 0.001). Negative emotions and attitudes towards aging are chain mediators of square dance exercise and quality of life, and the mediation consists of three pathways: separate mediation of negative emotions, separate mediation of attitudes towards aging, and chain mediation of negative emotions-attitudes towards aging.

**Conclusion:**

This study is dedicated to deepening the scientific role of how square dancing, as a health-promoting activity, can optimize the quality of life of middle-aged and older adults through the mediating mechanism of negative emotion alleviation and positive attitudes towards aging and then optimize the quality of life of middle-aged and older adults. This process not only enriches the theoretical framework of the complex interactions between psychosocial factors and healthy aging but also provides a solid empirical foundation for the development of health intervention strategies aimed at enhancing the overall well-being and quality of life of the middle-aged and older adults.

## 1 Introduction

The WHO estimates that from 2020 to 2030, the population aged 60 years or older will increase from 1 billion to 1.4 billion and that by 2050, the global population aged 60 years or older will more than double from 1 billion to 2.1 billion (1). Therefore, how to effectively improve the quality of life of older adults in the context of continued aging is not only related to the health and well-being of hundreds of millions of older adults at present but also one of the urgent tasks to achieve intergenerational harmony and maintain social vitality, as well as an important issue in the present and focus on the long term.

With the increasing population aging in China, the proportion of chronic disease patients and disabled and semidisabled older adults has increased annually. According to a survey report, at present, there are approximately 35 million disabled older adults in China, accounting for 11.6% of all older adults, and according to the estimation, the number of disabled older adults in China will reach 46 million in 2035 and approximately 58 million in 2050 (2). In addition, middle-aged and older adults face the pain of bereavement, the loss of social status, the gradual weakening of social relationships, and the psychological burden caused by older adults all of which affect their quality of life (QoL). Therefore, caring for the physical and mental health of middle-aged and older adults has become the key to promoting healthy aging.

Physical activity is globally recognized as a positive health asset for all ages and is a non-pharmacological intervention for the prevention and treatment of many diseases (3). Older adults who are physically inactive are more prone to various types of physical and psychological problems, such as poor balance and endurance qualities, cardiovascular disease, depression, anxiety, and stress, which reduce their quality of life (4). Therefore, it is particularly important to explore physical activity interventions. The Road to Healthy Aging in China: A Peking University Lancet Major Report presents the health benefits of social interaction and participation in leisure and recreational activities (e.g., square dancing) for older adults, demonstrating the positive effects of square dancing on older adults’ physical and mental health (5). Square dancing, as a form of exercise spontaneously organized by the masses and carried out in public spaces with music as the carrier, has become a very popular collective fitness and social activity in China, with a low cost of participation and easy-to-learn “low threshold” characteristics, and has been welcomed by an increasing number of middle-aged and older adults in China (6). Studies have estimated that more than 100 million people in China participate in recreational square dancing, with middle-aged and older adults being the main participants (7). Middle-aged and older adults in China choose to participate in square dancing because of the importance they place on social relationships and psychological values and because square dancing is a cluster-based physical and social activity that helps them expand their social circle and reduces their sense of loneliness, depression, and anxiety (8).

In addition, square dancing is often regarded as a representation of improved quality of life (9), and research on square dancing has been expanding from a physiological perspective to a psychological and social perspective, with positive effects on the physical and mental health of middle-aged and older adults, as well as an increase in social participation, which is beneficial to improving the level of quality of life (10, 11). The physiological effects of participating in square dance exercise include lowering body weight, improving balance, coordination, and endurance, and preventing the risk of falls in the older adult (12), and lowering the risk of cardiovascular disease in the older adult (13), and the psychological and social effects include increasing the subjective sense of well-being of the participants, enhancing social interactions (10), slowing down depressive moods, and promoting the formation of positive attitudes toward aging, etc. (14). Among them, depressed mood and attitudes towards aging are also the focus of this study.

Therefore, according to previous studies, square dance exercise may be used as an important means to promote the quality of life level of middle-aged and older adults, as well as to promote healthy aging, but the mechanism of association between square dance, as a special form of exercise for middle-aged and older adults in China, and their quality of life has not been fully confirmed. Although it has been confirmed in related studies that negative emotions (e.g., symptoms such as depression, anxiety, stress, etc.) and negative attitudes towards aging are important factors affecting quality of life, and some studies have shown that participation in square dancing is conducive to the reduction of negative emotions, prompts middle-aged and older adults to form positive attitudes towards aging, and that the longer the duration of participation lasts, the more positive attitudes toward one’s own aging will be (10). However, the direct effect of square dancing on the quality of life and the use of negative emotions and attitudes towards aging as mediating variables in the middle-aged and older adults have been less studied in the middle-aged and older adults. Therefore, in order to fill this research gap, this study takes negative emotions and attitudes towards aging as mediating variables between square dance exercise and quality of life in middle-aged and old-aged people from a psychological perspective, expecting to verify the chain mediating effects of negative emotions and attitudes towards aging through a large-sample cross-sectional study, clarify the relationship between square dance exercise and quality of life of middle-aged and old-aged people and its internal mechanism of action, and provide a healthy aging promotion research basis and theoretical reference for the promotion of healthy aging.

### 1.1 Square dance and quality of life

Quality of life (QoL) is defined as “an individual’s perceived position in life in the context of the culture and value system in which they live and in relation to their goals, expectations, standards and concerns.” In terms of subjective indicators, quality of life (QoL) reveals the state of people in terms of psychological, emotional and well-being and is an important component of the health status of older people (15).

Currently, relevant studies have confirmed the correlation between aerobic exercise and QoL, for example, square dance, yoga, social dance, and tai chi exercise interventions have small to moderate improvements in somatic function, quality of life, and mental health in people over 60 years of age (16, 17), which are inextricably intertwined, and relevant empirical studies have revealed a positive correlation between square dance exercise and QoL, with continued participation in square empirical studies revealing that there is a positive correlation between square dance exercise and quality of life and that a longer duration of continuous participation in square dance exercise is associated with the best perceived quality of life of older adult individuals (10), especially in improving the quality of life of middle-aged and older adult women (psychological dimension) (18). Square dance exercise can promote improvements in quality of life to a certain extent, and the enjoyment of a high level of quality of life is also one of the important goals of middle-aged and older adults to participate in square dance exercise (19). This leads to research hypothesis H1: Square dance exercise can positively predict the quality of life of middle-aged and older adult individuals.

### 1.2 Mediating role of negative emotions

Negative emotions are a series of unpleasant experiences in a state of high arousal, including emotions such as anxiety, depression, and stress (20). Studies have shown that there is a significant relationship between physical activity and depressive symptoms (21), that physical activity is positively associated with positive mental health in middle-aged and older women, and that aerobic exercise is recognized as a unique resource for positive mental health (22).

Emotional valence theory research suggests that positive and negative emotions trigger different judgments and decision-making behaviors in individuals, with positive emotions leading to optimistic judgments and negative emotions leading to pessimistic judgments (23); thus, positive emotions may increase the enthusiasm of middle-aged and older adults to participate in physical activity, and the positive effects of moderate physical activity on the negative emotions of middle-aged and older adults have already been verified (24), helping middle-aged and older adults make optimistic judgments, which in turn affects their quality of life. Compared with other forms of exercise, participation in regular aerobic exercise (e.g., square dancing) can improve the negative mood and mental state of middle-aged and older adult individuals, probably because aerobic exercise can prompt the body to secrete neurotransmitter-like chemicals that are more capable of reducing depression, anxiety and other negative emotions. Empirical studies have shown that the participation of people aged 45 years and above in group, recreational and social square dance exercise has a more significant effect on the negative emotions of middle-aged and older adults, especially in the improvement of depression, anxiety and other aspects, which can further improve the overall quality of life (25). Therefore, improving quality of life through square dance exercise is crucial for the prevention and management of chronic diseases such as depression and anxiety in middle-aged and older adult women. In addition, some studies have confirmed that negative psychological factors (e.g., depression, anxiety, and perceived stress) mediate the relationship between physical activity and quality of life (26) and that effectively reducing negative emotions may influence middle-aged and older adults perceptions of life satisfaction and explain the effects of physical activity on quality of life (27). As a result, research hypothesis H2 is proposed: negative emotions mediate the relationship between square dance exercise and quality of life.

### 1.3 Mediating role of attitudes towards aging

Attitudes towards aging refer to older adults’ attitudes toward their own aging process, which is an individual’s experience of perceiving his or her own aging as well as expectations about the results and process of becoming older (28). There are positive and negative attitudes toward aging: positive attitudes toward aging mean that experiences, feelings, and perceptions in the face of aging are positive and conducive to positive aging, whereas negative attitudes toward aging suggest that old age implies a loss of psychological, physical, and social aspects (29). The transition from middle age to old age is characterized by potentially dramatic life changes, such as bereavement and retirement, which may also contribute to the risk of depression in middle-aged and older adults as they age.

The “Successful Aging Selective Optimization with Compensation (SOC) Model” emphasizes that middle-aged and older adults, when faced with the loss of physical, psychological, and social resources, should identify and focus on the most valuable areas of activity, optimize the allocation of resources, and adopt compensatory strategies to minimize the impact of loss. This model encourages older adults to actively manage resources to continue taking on valued roles and activities that enhance life satisfaction and well-being (30). Square dance exercise has been recognized as an important expression of providing middle-aged and older adults with the opportunity to participate in groups that allow them to experience social relationships (8). In addition, studies have demonstrated that physical activity can help middle-aged and older adults achieve good physical function and positive emotional experiences, prevent disease, promote mental health, and effectively improve attitudes toward aging (31). Moreover, sports activities involve the idea of social participation in “learning, enjoying and doing something for the older adult” (32). Together, these findings suggest that participation in square dance exercise helps middle-aged and older adults form a positive attitude toward aging, thus improving their quality of life.

In recent years, empirical studies on middle-aged and older adults have shown that physical exercise can promote positive attitudes toward aging and that attitudes towards aging can significantly affect their mental health (33), which in turn affects their quality of life, confirming the close correlation between the attitudes towards aging of middle-aged and older adults and their quality of life. In addition, attitudes toward aging can significantly predict the level of quality of life in middle-aged and older adult individuals, and positive attitudes toward aging can promote their high quality of life. In this study, square dance exercise effectively reduced the dimension of psychosocial loss, improved the dimension of physical change, enhanced the sense of psychological gain, and further improved the quality of life of middle-aged and older adult individuals (34, 35). In view of this, research hypothesis H3 is proposed: attitudes towards aging mediate the relationship between square dance exercise and quality of life.

### 1.4 Chain mediation of negative emotions and attitudes towards aging

Among middle-aged and older adults, square dance is the main way for Chinese middle-aged and older adults to participate in physical exercise and one of the ways for middle-aged and older adults to participate in social activities (7). Participating in square dance exercise is an effective way for middle-aged and older adults to adapt positively to aging, which can enhance individual well-being, reduce negative emotions, and improve the quality of life of middle-aged and older adults. On the one hand, improved mood can produce more positive attitudes toward aging; on the other hand, depressed mood may have a significant effect on attitudes towards aging by activating negative aging stereotypes (36). When middle-aged and older adults have more positive feelings in the face of self-aging, it can lead to higher levels of life satisfaction and subjective well-being (37), whereas negative attitudes towards aging are predicted to lead to more depression and anxiety (38), resulting in a greater emotional response to daily stressors (39). On the other hand, some studies have shown that there is a correlation between attitudes towards aging and negative emotions, and both physical change attitudes and psychological growth attitudes of middle-aged and older adults are negatively correlated with depression. In addition, square dance exercise has a good predictive effect on reducing psychosocial loss and forming positive physical change attitudes in middle-aged and older adults (38). Therefore, positive attention should be given to attitudes towards aging, and improving attitudes towards aging may help reduce the burden of depression, anxiety, and perceived stress in middle-aged and older adults (40).

In summary, negative emotions and attitudes towards aging are likely to be important mediating variables between square dance exercise and quality of life. This relationship has yet to be further validated, particularly for the middle-aged and older adult populations. Thus, the present study proposes research hypothesis H4: Negative emotions and attitudes towards aging play a chain mediating role between square dance exercise and quality of life.

### 1.5 The present study

**Figure 1:**
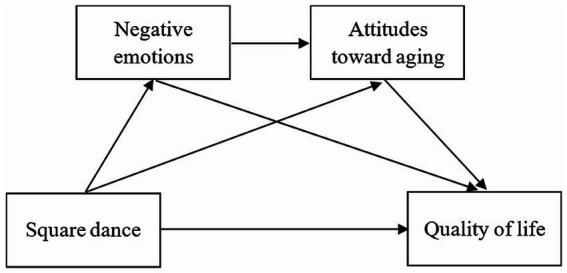
Research hypothesis model.

Previous research on square dance exercise has focused on the effects of square dance exercise on the physical and mental health of older adults (11); however, the role of square dance exercise throughout the transition from middle age to old age also deserves attention. The present study aimed to explore the mechanism underlying the association between square dance exercise and quality of life in a core group of Chinese middle-aged and older adults to fill in the gaps of previous studies and to provide new ideas for aging interventions (Figure 1). Therefore, the research hypotheses of this study are as follows:

*H1*: Square dance exercise positively predicts quality of life in middle-aged and older adult individuals.

*H2*: Negative emotions mediate the relationship between square dance exercise and quality of life.

*H3*: Attitudes towards aging mediate the relationship between square dance exercise and quality of life.

*H4*: Negative emotions and attitudes towards aging chain-mediate the relationship between square dance exercise and quality of life.

## 2 Materials and methods

### 2.1 Participants

A total of 5,714 square-dancing participants were recruited online by Wenjuanxing^1^ from 19 August to 12 September 2023 in China. We monitored the IP addresses of the respondents to avoid multiple responses. The questionnaire was distributed across 30 provinces, autonomous regions, and municipalities, yielding a wide geographical distribution of the sample. Including Zhejiang, Beijing, Jiangsu, Guangdong, Fujian, Inner Mongolia, Shanghai, Qinghai, Shandong, Yunnan, Hunan, and Chongqing.

Since the data were self-reported by the participants, outliers may exist due to input errors or recall bias. To minimize the impact of outliers on data accuracy, SPSS 27.0 software was used to clean the data against the scale. On the basis of the analysis of the response time of middle-aged and older adults and the total number of questions, five aspects were excluded: the time taken was too short (the average time taken for each question was less than 2 s), not all the questions were answered (missed questions), the age did not comply with the regulations (less than 45 years old), the number of the same options in the whole volume of the response was greater than 70%, and the lie detector mention was filled in incorrectly (41). Finally, 4,636 valid questionnaires were recovered, with an effective rate of 82%. The age range of the association members was 45–75 years, with an average age of 59.08 ± 7.56 years. Among them, 213 (4.6%) were male, and 4,423 (95.4%) were female.

### 2.2 Measures

#### 2.2.1 Basic demographic information

The demographic variables include age, sex, highest level of education, employment status, marital status, monthly income, intensity and frequency of square dance exercise, square-dancing group size and square-dancing exercise duration.

#### 2.2.2 Square dance exercise

Square dance exercise was assessed via the Physical Activity Rating Scale-3 (PARS-3), a questionnaire used to examine an individual’s actual participation in square dance exercise in the most recent month. In this study, the Chinese version translated and revised by Liang Deqing was used to conduct the survey (42). The questionnaire contains three topics, namely, exercise intensity, duration, and frequency of activity, and the scale is scored on a 5-point scale, with the scoring method of exercise = intensity × duration × frequency, with the maximum score being 100 and the minimum score being 0. A higher score indicates a greater amount of exercise. A score of less than or equal to 19 on the scale is considered small exercise, a score of greater than 19 and less than or equal to 42 is considered moderate exercise, and a score of greater than or equal to 43 is considered large exercise. The Cronbach’s *α* of the scale was 0.65, and the retest reliability of the items was 0.83. In addition, we added validity test (EFA), yielding a KMO coefficient of 0.86.

#### 2.2.3 Negative emotions

Negative mood was measured via the Depression Anxiety and Stress Scale (DASS-21). The scale includes 3 subscales of depression, anxiety and stress, totaling 21 items, and each subscale contains 7 items. A 4-point scale (0 = not at all, 1 = partially, 2 = mostly, 3 = fully) was used to assess the individual’s negative mood symptoms in the past 1 week, with higher scores indicating more intense negative mood experiences. Moreover, the Cronbach’s coefficient of the total scale was 0.912, the Cronbach’s coefficient of the subscales ranged from 0.754–0.823, and the validity coefficient of the dimensions ranged from 0.895–0.910, which meets the requirements of measurement science and has good applicability and rigor (43). In this study, the Cronbach’s *α* of the DASS-21 was 0.91.

#### 2.2.4 Attitudes toward aging

Attitudes toward aging were assessed via the Attitudes to Aging Questionnaire (AAQ) (44), which has 3 dimensions with 8 entries per dimension, for a total of 24 entries. The psychosocial loss (Psysoloss) subscale focuses on older adults’ attitudes toward aspects of psychological and social loss. For example, “Old age is a frustrating time of life” and “I do not feel included in society when I am older” The Physical Changes (Phych) subscale assesses older adults’ attitudes toward physical functioning, with items related primarily to experiences of health, exercise and aging. Examples include “Exercise is important at any age” and “I have more energy now than I expect for my age” The Psychological Growth (Psygro) subscale reflects older adults’ positive feelings of acquisition in relation to self and in relation to others. For example, “I am more accepting of myself as I age” and “I want to set a good example for younger people” Each item was rated on a five-point Likert scale, with 1 being “not true at all” and 5 being “completely true” Scores for each of the three dimensions were obtained by summing the corresponding items (45). Phych and Psygro are positive dimensions, with higher scores indicating more positive attitudes toward aging, whereas Psysoloss is a negative dimension, with more scores indicating more negative attitudes toward aging. In this study, the Cronbach’s *α* of the AAQ was 0.93.

#### 2.2.5 Quality of life

Quality of life was measured via the 12-Item Short Form Health Survey (SF-12), which is primarily used to assess the impact of health on an individual’s daily life. This instrument has been widely used in a variety of populations (46). The SF-12 measures 8 dimensions of health, namely, general health, physical functioning, somatic functioning in role functioning, somatic pain, mental health, emotional impact on role functioning, vitality, and social functioning (47), and has demonstrated good reliability and validity in a variety of populations (48). The first four dimensions were used to assess physical health (PCS), whereas the last four dimensions were used to assess mental health (MCS). The Cronbach’s alpha coefficient for the SF-12 across the eight dimensions was 0.79, indicating relatively high internal consistency across items. Higher SF-12 scores indicate better quality of life (49). In this study, the Cronbach’s *α* for QoL was 0.86.

### 2.3 Statistical analysis

SPSS 27.0 and Mplus 8.3 were used to organize and analyze the data in this study. First, SPSS 27.0 was used to organize the collected data, perform descriptive statistics and correlation analysis, and test the reliability and validity of all the scales; second, Mplus 8.3 was used to construct a structural equation model for analysis and to test the model fit and mediation effects.

## 3 Results

### 3.1 Common method bias test (CMV)

The data in this study were obtained from self-reports, which may lead to the issue of common method bias. Therefore, this study made necessary controls during the measurement process, such as protecting the anonymity of the subjects, stating to the subjects in the informed consent form that the data obtained were used only for scientific research, using reverse scoring for some of the questions in the questionnaire, and so on, in addition to using Harman’s one-factor test for statistical control before the data were analyzed, and non-rotated principal component factor analysis was performed on all the variable items. The results revealed that the cumulative explained variance of the first factor in this study was 35.49%, which is less than the critical value of 40% (50). Therefore, there is no serious common method bias problem in this study.

### 3.2 Descriptive statistics

Table 1 shows the demographic information, means and standard deviations of the participants’ age, sex, highest level of education, employment status, marital status, monthly income, intensity and frequency of square dance exercise, square-dancing group size and square-dancing exercise duration.

**Table 1:** Demographic information of the participants.

Sociodemographic characteristic	*M(SD)* or *N*(%)
Age (years)	59.08 ± 7.56
Sex	
Female	4,423 (95.4%)
Male	213 (4.6%)
Highest level of education	
Primary school	306 (6.6%)
Junior middle school	1,628 (35.3%)
Senior middle school	1,687 (36.3%)
Bachelor’s degree or above	1,015 (21.8%)
Employment status	
Employed	872 (18.8%)
Retired	3,764 (81.2%)
Marital status	
Married	4,389 (94.6%)
Unmarried	247 (5.4%)
Monthly income	
<3,500 yuan	2,343 (50.5%)
>3,500 yuan	2,293 (49.5%)
Square-dancing exercise intensity	17.35 ± 12.35
Light exercise	924 (19.9%)
Small intensity	2,723 (58.7%)
Medium-intensity	890 (19.2%)
High-intensity (not long-lasting)	77 (1.6%)
High-intensity (long-lasting)	22 (0.6%)
Square-dancing exercise Frequency	
Less than 1 time a month	302 (6.3%)
3–5 times a week	266 (5.5%)
2–3 times a month	1,413 (29.3%)
Approximately 1 time per day	1,048 (21.7%)
1–2 times a week	1,607 (33.3%)
Square-dancing group size	
<25 people	2,460 (53.0%)
>25 people	2,176 (47.0%)
Square-dancing exercise duration	
<5 years	2021 (43.6%)
>5 years	2,615 (56.4%)

M, mean; SD, standard deviation; N = absolute frequency; % = relative frequency.

As shown in Table 1, the mean age of the 4,636 study participants was 59.08 ± 7.56 years. Among them, 4,423 females accounted for 95.4% of the total number, and 213 males accounted for 4.6% of the total number. In terms of education level, 1,015 people had a bachelor’s degree or above, accounting for 21.8% of the sample; 1,687 had a senior middle school education, accounting for 36.3%; 1,628 had a junior middle school education, accounting for 35.3%; and 306 had a primary school education, accounting for 6.6%. Currently, 3,764 (81.2%) are retired (about to retire), and 872 (18.8%) are still working. Marital status was married (4,389), or 94.6% of the total, and divorced (247), or 5.4% of the total. The monthly household income was relatively average, with 2,343 reporting a monthly income of less than <3,500 yuan and 2,293 reporting a monthly income of >3,500 yuan, accounting for approximately 50% of the total.

In terms of square dance exercise intensity, small-intensity exercise accounted for 58.7% of all participants, in addition to focusing on light exercise and medium-intensity exercise, accounting for 39.1% of the participants; in terms of square dance exercise frequency, the participants who participated in square dance exercise 1–2 times a week the most (33.3%), followed by 2–3 times a month (29.3%), and approximately 1-time exercise per day (21.7%). In terms of the number of years of square dance exercise, the number of participants who had participated in square dance exercise for less than 5 years and more than 5 years tended to be the same.

### 3.3 Correlation analysis

The mean, standard deviation, and Pearson correlation matrix for each variable are presented in Table 2. Correlation analysis revealed that square dance exercise was significantly correlated with negative emotions, attitudes towards aging, and quality of life (*r* = −0.042–0.878, *p* < 0.01). Further correlation analysis of the dimensions of the scale revealed that, except the psychosocial loss dimension of attitudes towards aging, which was not significantly correlated (*p* > 0.05), the remaining dimensions were as follows: square dance exercise was significantly negatively correlated with negative emotions (*r* = −0.092–0.878, *p* < 0.01); significantly positively correlated with attitudes toward aging (*r* = −0.004–0.634, *p* < 0.01); and significantly positively correlated with quality of life (*r* = −0.394–0.280, *p* < 0.01). Negative emotions were significantly correlated with attitudes toward aging (*r* = 0.153–0.878, *p* < 0.01). Negative emotions (*r* = −0.394–0.878, *p* < 0.01) and attitudes toward aging (*r* = −0.394–0.634, *p* < 0.01) were significantly correlated with quality of life.

**Table 2:** Descriptive statistics and associations among the variables (*N* = 4,636).

Variables	*M*	*SD*	1	2	3	4	5	6	7	8
1. PARS-3	17.35	13.25	–							
2. DASS-Depression	18.35	7.28	−0.085**	–						
3. DASS-Anxiety	19.24	7.36	−0.092**	0.878**	–					
4. DASS-Stress	20.79	7.87	−0.073**	0.849**	0.871**	–				
5. AAQ-Psychosocial loss	28.79	7.79	0.004	0.018	0.022	0.019	–			
6. AAQ-Physical change	31.65	6.32	0.156**	−0.153**	−0.127**	−0.099**	−0.035**	–		
7. AAQ-Psychological growth	26.41	6.14	0.071**	0.093**	0.115**	0.113**	−0.004	0.634**	–	
8. SF-PCS	47.00	8.83	0.165**	−0.233**	−0.273**	−0.235**	−0.080**	0.277**	0.123**	–
9. SF-MCS	52.67	10.06	0.108**	−0.394**	−0.390**	−0.379**	−0.042**	0.280**	0.028	0.055**

**p < 0.01.

### 3.4 Differential analysis

By incorporating control variables such as sex, duration, and group size, we further explored the variability of each variable among the middle-aged and older adult groups, and the results of the independent sample *t*-test are as follows. (1) There was a significant difference between males and females in terms of square dance exercise (*p* < 0.001), and the level of male PA was greater than that of female PA. (2) There was a significant difference between the four aspects of square dance exercise, negative emotion, attitudes towards aging, and quality of life (*p* < 0.001). Compared with square dance participants with less than 5 years of participation and fewer than 25 group sizes, square dance participants with more than 25 group sizes had higher levels of PA, older attitudes, lower levels of negative emotion, and quality of life. The specific values are shown in Table 3.

**Table 3:** Independent samples *t*-test for each variable (*N* = 4,636).

Variables	Classification	Square dance exercise (*M* ± *SD*)	*t*	Negative emotion (*M* ± *SD*)	*t*	Attitudes towards aging (*M* ± *SD*)	*t*	Quality of life (M ± S*D*)	*t*
Sex	Male	18.10 ± 12.29	0.400	54.42 ± 17.61	0.006**	90.73 ± 12.69	<0.001***	103.74 ± 15.12	<0.001***
	Female	17.32 ± 13.30		58.56 ± 21.64		86.67 ± 13.57		98.21 ± 19.34	
Duration	<5 years	15.96 ± 12.41	<0.001***	53.36 ± 17.30	<0.001***	85.31 ± 12.90	<0.001***	92.12 ± 19.27	0.000***
	>5 years	18.43 ± 13.77		64.87 ± 24.43		88.05 ± 13.92		107.25 ± 16.79	
Group size	<25 people	16.07 ± 12.35	<0.001***	63.43 ± 23.53	<0.001***	85.36 ± 12.84	<0.001***	91.96 ± 19.21	<0.001***
	>25 people	18.80 ± 14.06		52.67 ± 17.20		88.55 ± 14.14		107.84 ± 15.26	

**p < 0.01, ***p < 0.001.

### 3.5 Mediating effect analysis of negative emotions and attitudes towards aging

In this study, a bootstrap mediation effect test was conducted on negative emotions and attitudes towards aging, with square dance exercise as the independent variable, negative emotions and attitudes towards aging as the mediating variables, and quality of life as the dependent variable. The sample size was set at 5,000, the confidence interval was 95%, and the bootstrap method was the bias-corrected non-parametric percentile method. Before the mediating effect was tested, the direct effect of square dance exercise on quality of life was first tested, and the model fit index was found to be good, χ^2^/*df* = 7.82, CFI = 0.976, TLI = 0.939, RMSEA = 0.038, SRMR = 0.015. The results revealed a significant positive predictive effect of square dance exercise on quality of life (*β* = 0.878, *p* < 0.001).

Next, Model 1 and Model 2 were established with negative emotions and attitudes towards aging as single mediating variables, respectively. The results showed that Model 1 and Model 2 fit better (Model 1: χ^2^/*df* = 9.49, CFI = 0.994, TLI = 0.986, RMSEA = 0.043, SRMR = 0.025; Model 2: χ^2^/*df* = 23.09, CFI = 0.936, TLI = 0.897, RMSEA = 0.069, SRMR = 0.032). The bias-corrected bootstrap method was used to repeat the sampling 5,000 times for the test of mediating effects. The mediating effect value for negative emotions was 0.126. 95% CI = [0.089, 0.162], excluding 0, with an effect size of 28.1%. The mediated effect value for attitudes toward aging was 0.171, 95% CI = [0.142, 0.200], excluding 0, with an effect size of 28.2%.

Next, the Model 3 chain mediation model was developed. The results showed a good fit, with fit indices of χ^2^/*df* = 23.33, CFI = 0.965, TLI = 0.950, RMSEA = 0.069, SRMR = 0.053. The individual path coefficients were as follows: (1) square dance exercise significantly and positively predicted quality of life (*β* = 0. 202, *p* < 0.001); (2) square dance exercise significantly and negatively predicts negative affect (*β* = −0.153, *p* < 0.001); (3) square dance exercise significantly positively predicted attitudes towards aging (*β* = 0.214, *p* < 0.001); (4) negative emotions significantly negatively predicted quality of life (*β* = −0.687, *p* < 0.001), and attitudes towards aging significantly positively predicted quality of life (*β* = 0. 481, *p* < 0.001); and (5) negative emotions significantly negatively predicted attitudes towards aging (*β* = −0. 100, *p* < 0.001).

**Figure 2:**
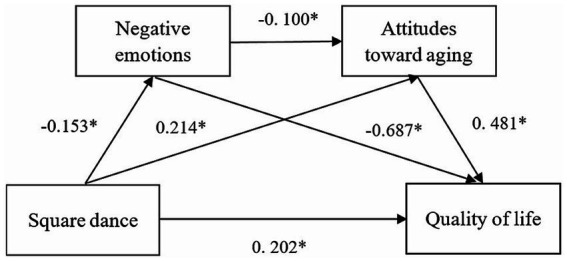
Chain mediating effect model of negative emotions and attitudes towards aging between square dance exercise and quality of life.

The bias-corrected bootstrap method was used to test the chained mediation model for each pathway situation with 5,000 repeated samples. Table 3 shows that the total effect of square dance exercise on quality of life was 0.417 with a 95% CI of [0.359, 0.475], excluding 0, and the total effect was significant. The direct effect was 0.202 excluding 0 with a 95% CI of [0.151, 0.254] excluding 0 and a significant direct effect. The mediating effect for negative emotions was 0.105 with a 95% CI of [0.074, 0.136], excluding 0, a significant mediating effect, and an effect size of 25.18%. The mediating effect for attitudes toward aging was 0.103 with a 95% CI of [0.083, 0.125], excluding 0, a significant mediating effect, and an effect size of 24.70%. The chained negative emotion and attitudes towards aging mediated effect was 0.007, and the 95% CI was [0.005, 0.011] excluding 0. A chain mediation was established, and the effect size was 1.68%. The above results indicate that negative emotions and attitudes towards aging act as chain mediators between square dance exercise and quality of life. Hypothesis 4 is verified. The total, direct and indirect effects are detailed in Figure 2 and Table 4.

**Table 4:** Bias-corrected bootstrap results for each path coefficient of the model.

Intermediary process	Effect value	Bootstrapped LLCI^a^	Bootstrapped ULCI^a^	Effect size (%)
Square dance exercise → negative emotions → quality of life	0.105	0.074	0.136	25.18
Square dance exercise → attitude towards aging → quality of life	0.103	0.083	0.125	24.70
Square dance exercise → negative emotions → attitude towards aging → quality of life	0.007	0.005	0.011	1.68
Direct effect	0.202	0.151	0.254	48.4
Indirect effect	0.215	0.178	0.252	51.5
Total effect	0.417	0.359	0.475	100

^a^Boot LLCI and Boot ULCI refer to the 95% confidence intervals of the indirect effect estimated via the bias-corrected bootstrap method and refer to the upper and lower limits of the intervals, respectively. CI, confidence interval. Total effect = indirect effect + direct effect.

Further analysis of the mediating effects revealed that bootstrap 95% confidence intervals did not include a value of 0 for the total indirect effect produced by negative emotions and attitudes towards aging, indicating that there was a significant mediating effect of negative emotions and attitudes towards aging between square dance exercise and quality of life. As shown in Table 4, the direct effect was 48.4% and the indirect effect was 51.5%. Square dance exercise → negative emotions → quality of life, 25.18%; square dance exercise → attitudes towards aging → quality of life, 24.70%; and square dance exercise → negative emotions → attitudes towards aging → quality of life, 1.68%.

## 4 Discussion

As a popular leisure sport among Chinese middle-aged and older adults, the positive effects of square dancing on physical and mental health echo previous findings on physical activity and attitudes towards aging, enhancing middle-aged and older adults sense of belonging and sense of community (8). Studies have shown that square dancing not only improves body coordination, flexibility, and lower limb strength but also that these body strengthening effects are consistent with the findings of yoga methods (51). In addition, square dancing, as a group activity, significantly improves the quality of life of middle-aged and older adults by enhancing their sense of belonging and providing opportunities for social interaction, which is consistent with the findings of taijiquan in promoting social interaction and improving quality of life (52).

On the basis of emotional utility theory and the successful aging model-selective compensation optimization (SOC) model, this study used cross-sectional survey methods to systematically explore the association mechanisms between participation in square dance exercise and quality of life in middle-aged and older adults, as well as the mediating effects that negative emotions and attitudes towards aging play in the effects of square dance on quality of life. Square dance exercise enhances social interactions by reducing the probability of negative emotions, which in turn promotes the formation of positive attitudes toward aging. In addition, this study reveals the positive effect of square dance exercise on quality of life, while the chain mediation model is finally formed under the premise that the individual mediators are all valid, and the results validate H1, H2, H3, and H4.

### 4.1 Direct effects of square dance exercise on the quality of life

Square dance exercise has a significant positive effect on the quality of life of middle-aged and older adults, and participation in square dance exercise is conducive to improving quality of life. Maintaining a continuous and appropriate amount of square dance exercise not only promotes human metabolism and stabilizes the physiological functions of the human body but also improves the mental outlook, enhances the vitality of the human body, and improves the self-confidence of life of middle-aged and older individuals, which is in agreement with the results of a previous study (53). As a unique form of activity among middle-aged and older adults, square dance exercise, specifically social interaction, distinctive rhythmic elements, and appropriate physical intensity are the keys to differentiating it from other forms of exercise (54), and these features work together to promote the physical and mental health of participants. First, the social interaction of square dances provides an important communication platform for older adult individuals. In the process of dancing together, they can not only make new friends but also share their lives in a relaxed and happy atmosphere, and this positive social interaction helps reduce negative emotions and enhances their sense of social belonging, which in turn generates a positive attitude toward aging and enhances their quality of life.

Second, the rhythmic element of square dance is its unique charm. With the rhythmic changes in music, participants are able to immerse themselves in rhythm and melody, and this sense of rhythm not only stimulates the body’s natural motor response and improves body coordination but also promotes the activity of the cerebral cortex, which contributes to the maintenance and improvement of cognitive functions (55). At the same time, rhythmic movement can also trigger positive emotional experiences, such as pleasure and fulfillment, further enhancing emotional states. Finally, the results of the study revealed that the exercise intensity of many square dance participants was low, which is suitable for assessing the physiological characteristics of middle-aged and older adult individuals. It is not too strenuous to avoid causing sports injuries; it can also effectively promote the body’s metabolism and stabilize physiological functions. Continuous square dance exercise can enhance cardiorespiratory function, improve muscle strength and endurance, and reduce the risk of chronic diseases, thus improving overall physical health. The improvement in physical health is then reflected in the state of mental health, which is conducive to middle-aged and older adults showing greater vitality and vigor and producing a positive level of quality of life (56, 57).

Thus, it is further confirmed through social interaction, rhythmic elements, and exercise intensity aspects of square dancing that square dancing has a strong positive impact on the quality of life of middle-aged and older adult individuals.

### 4.2 Mediating effect of negative emotions

Negative emotions play a mediating role in the effects of square dance exercise on the quality of life of middle-aged and older adult individuals. Square dance exercise significantly and negatively predicts negative emotion, and negative emotion significantly and negatively predicts quality of life; thus, negative emotion plays a mediating role between square dance exercise and quality of life. Numerous studies have shown that actively engaging in regular physical exercise can lead to a significant decrease in depression levels; that physical exercise has a positive impact on physical, mental, and emotional health (17); and that a reduction in negative emotions such as depression, anxiety, and stress is conducive to improving quality of life in middle-aged and older adult individuals (58).

According to the neurobiological hypothesis, physical exercise can stimulate monoamine neurotransmitters, which can reduce depression, anxiety, and stress (59). Long-term physical exercise, especially aerobic exercise, can increase the expression of brain-derived neurotrophic factors and their receptors, which in turn reduces hippocampal atrophy, improves memory function, and reduces depression (60, 61). With age, along with atrophy of the hippocampus and other neural parts, the physiological functions of the middle-aged and older adult population change, and individuals gradually shift to relying on physical exercise to promote physical and mental health. A meta-analysis further confirmed that time spent exercising per week had a positive effect on reducing depressive symptoms and that the more time spent exercising per week, the more significant the reduction in depressive symptoms (62). Our findings showed that middle-aged and older adults who had participated in square dancing for more than 5 years had lower negative affect than those who had participated for less than 5 years did, suggesting that square dance exercise is beneficial in alleviating negative affect in middle-aged and older adults. This result further confirms research hypothesis 2: by participating in square dance exercise, a spontaneous and organized cluster of aerobic exercise, middle-aged and older adult individuals can also improve negative emotions such as depression, anxiety, and stress and gain positive emotional experiences while maintaining their physical abilities, thus improving the quality of life of middle-aged and older adult individuals.

### 4.3 Mediating effect of attitudes towards aging

Attitudes towards aging play a mediating role in the effects of square dance exercise on the quality of life of middle-aged and older adults. Square dance exercise significantly and positively predicted attitudes towards aging, and attitudes towards aging significantly and positively predicted quality of life. This finding is consistent with the results of many current studies (34, 63). Positive attitudes towards aging can improve quality of life in middle-aged and older populations. According to the successful aging model-selective compensation optimization (SOC) model, individuals have the potential to grow and be malleable during their aging process, although they may experience a reduction in various resources or losses in physical, psychological, and social domains. This dynamic equilibrium between gain and loss can be achieved through the interaction of three components: selection, compensation, and optimization. The gradual increase in age in the middle-aged and older adult population is accompanied by a decrease in organic health, social relationships and other social resources, as well as a decline in positive emotional experiences. Square dance exercise can provide internal and external resources to meet the needs of self-actualization in middle-aged and older adult groups (30), and the results of the present study revealed that attitudes towards aging, in which the psychosocial loss dimension does not have a correlation, may be related to the fact that, for example, middle-aged and older adults have found new social support and emotional connections in square dance exercise, which effectively alleviates potential psychosocial loss. Second, in the process of participating in square dance exercise, middle-aged and older adult individuals may compensate for or mitigate psychosocial loss through positive social interaction, psychological adjustment and behavioral change, which enhances their sense of self-worth and well-being and thus maintains a high quality of life.

Social–emotional choice theory further explains how middle-aged and older adults cope with psychosocial losses in the aging process through the choice of social goals and emotional management. As the perceived “end” of future time approaches, middle-aged and older adults are more likely to pursue positive emotional states and show “positive effects” in their emotional management. Relevant studies have confirmed that, from middle age to old age, attitudes towards aging generally become more positive (64) and can lead to stable emotional experiences, thus maintaining positive attitudes toward life, further promoting the participation of middle-aged and old-aged groups in square dance exercise as a social group activity, forming a physiological–psychological health cycle of mutual benefit, and further improving quality of life.

### 4.4 Chain-mediated effects of negative emotions and attitudes toward aging

The chain-mediated effect of square dance exercise on the quality of life of middle-aged and older adults through negative emotions and attitudes towards aging was verified. This finding is consistent with the results of previous studies (65), which suggest that physical activity has a positive effect on improving mood states and quality of life in older adults. In particular, square dancing, as a group physical activity, not only reduces depressive symptoms and improves quality of life in older adults (15) but also contributes to positive attitudes towards aging by reducing negative emotions. This finding echoes existing research suggesting that positive emotional experiences promote older adults’ participation in more social activities, thereby improving their quality of life (66).

Square dancing provides a platform for social interaction, which may be a unique advantage over studies of middle-aged and older adults’ participation in other forms of physical activity. For example, while exercises such as yoga and tai chi have also been shown to improve mental health and quality of life in older adults (67, 68), they are often viewed as more individualized activities. The communal nature of square dancing may be more effective in promoting social interactions and positive emotional experiences, which can contribute to the development of positive attitudes toward aging and, in turn, improve quality of life.

Additionally, social motivational choice theory emphasizes the tendency of older adults to remain connected to close partners. Square dance exercise provides an opportunity for older adults to develop stable and intimate relationships with team members over the course of long-term participation, resulting in more positive emotional experiences within the team. This increased social interaction, which is closely related to an individual’s positive emotional experience, is a key factor in forming positive attitudes towards aging and promoting successful aging mechanisms. Overall, square dance exercise positively impacts the quality of life of middle-aged and older adults by reducing negative emotions and enhancing social interactions and positive emotional experiences. This finding is not only consistent with previous findings on physical activity and quality of life in older adults (10) but also further emphasizes the potential value of square dancing in promoting positive attitudes towards aging and successful aging.

## 5 Limitations and recommendations

### 5.1 Limitations

This study has several limitations. First, the cross-sectional survey lacked long-term follow-up, and the data were derived from self-reports, which could lead to potential hypothesis bias. In addition, although previous research provided some foundation for this study, it is difficult to infer the characteristics of the variables over time and their causal relationships. More empirical studies, cross-lagged designs, and longitudinal tracking are needed to explore this model theory in the future to improve it. Second, the sample for this study was obtained through convenience sampling, which may limit the generalizability of the findings. There was also a gender imbalance in the sample, with the majority of participants being female, which may have affected the overall understanding of the impact of square dancing. Future research should consider the use of random sampling methods and ensure that the sample is balanced in terms of gender to assess the impact of square dancing on the quality of life of the middle-aged and older adult population more accurately. Finally, at the objective level, technical measures can be introduced to further observe the benefits of square dance development, such as the introduction of wearable technology (e.g., wearable fitness trackers, real-time monitoring of heart rate, step count, calorie consumption, and other key indicators, to provide a basis for personalized exercise plans); increasing community management and platform co-construction (development of apps or applets suitable for square dance, providing functions such as music playback, exercise guidance, health monitoring, etc., to enhance the exercise of the older adult); and increasing the use of community management and platform co-construction. Monitoring and other functions to enhance the exercise experience of older adult individuals.

### 5.2 Recommendations

(1) The results of the study show that negative emotions and attitudes towards aging are two important mediating variables in square dance exercise and quality of life. This finding shows that middle-aged and older adults can participate in square dance as a form of cluster exercise; pay attention to the improvement of the negative emotions of individuals through square dance exercise, i.e., it has a significant effect on reducing depression, anxiety, and stress; and, at the same time, pay attention to the formation of positive attitudes towards aging of individuals, pay attention to the subjective experience of individuals in terms of quality of life, and improve the degree of satisfaction with life and subjective well-being so that the relationship hierarchy of “square dance exercise—improving negative emotions—forming positive attitudes towards aging—improving individual quality of life” is effectively formed. Exercise—improving negative emotions—forming positive attitudes towards aging—improving individual quality of life and popularizing it. In addition, on the basis of the development of physical and mental health, the square dance organization group should pay attention to the dose effect of square dance exercise and regulate and guide the popular repertoire, exercise frequency and intensity, and single-time exercise to guarantee the intervention effect of square dance exercise, which will lead to an increasing number of middle-aged and older adults to participate in it and positively respond to the new trend of the global sports boom.

(2) At present, the square dance program has gradually formed a complete competition rule and promotion system and has been popularized and promoted throughout the country, and how to carry out the square dance program, which is mainly recreational and leisure for the general public, in a more efficient and high-quality manner, requires the joint efforts of senior citizen associations, social organizations, square dance organizations, and other social forces to formulate workout repertoires and styles suitable for the general population and to attract more people to participate in the program. At the same time, accounting for the factors of chronic diseases and special people, we will create a dance repertoire suitable for “integration of the disabled and the healthy” to create a good atmosphere for the integration of the disabled and the healthy, to create a good social culture of helping the disabled and the handicapped, to basically realize the full coverage of all kinds of people to participate in the square dance program, to pay attention to the physical and mental development of individuals, to achieve the goal of physical exercise, and to make the program more effective and efficient. At the same time, it pays attention to the physical and mental development of individuals and achieves the goal of promoting mental health through physical exercise.

## 6 Conclusion

This study is dedicated to deepening the scientific role of how square dancing, as a health-promoting activity, can optimize the quality of life of middle-aged and older adults through the mediating mechanism of negative emotion alleviation and positive attitudes towards aging and then optimize the quality of life of middle-aged and older adults. This process not only enriches the theoretical framework of the complex interactions between psychosocial factors and healthy aging but also provides a solid empirical foundation for the development of health intervention strategies aimed at enhancing the overall well-being and quality of life of the middle-aged and older adults.

## Data Availability

The raw data supporting the conclusions of this article will be made available by the authors, without undue reservation.

## Footnotes

^1^ https://www.wjx.cn/
